# IL-7: A promising adjuvant ensuring effective T cell responses and memory in combination with cancer vaccines?

**DOI:** 10.3389/fimmu.2022.1022808

**Published:** 2022-10-28

**Authors:** Yue Zhao, Kongyuan Wei, Hao Chi, Zhijia Xia, Xiaosong Li

**Affiliations:** ^1^ Cancer Hospital of the University of Chinese Academy of Sciences (Zhejiang Cancer Hospital), Institute of Basic Medicine and Cancer (IBMC), Chinese Academy of Sciences, Hangzhou, Zhejiang, China; ^2^ Department of General, Visceral, and Transplant Surgery, Ludwig-Maximilians-University Munich, Munich, Germany; ^3^ Department of General, Visceral and Transplantation Surgery, University of Heidelberg, Heidelberg, Germany; ^4^ Clinical Medical Collage, Southwest Medical University, Luzhou, China; ^5^ Clinical Molecular Medicine Testing Center, The First Affiliated Hospital of Chongqing Medical University, Chongqing, China

**Keywords:** IL-7, adjuvant, cancer vaccines, T cell immune response, T cell memory

## Abstract

Cancer vaccines exhibit specificity, effectiveness, and safety as an alternative immunotherapeutic strategy to struggle against malignant diseases, especially with the rapid development of mRNA cancer vaccines in recent years. However, how to maintain long-term immune memory after vaccination, especially T cells memory, to fulfill lasting surveillance against cancers, is still a challenging issue for researchers all over the world. IL-7 is critical for the development, maintenance, and proliferation of T lymphocytes, highlighting its potential role as an adjuvant in the development of cancer vaccines. Here, we summarized the IL-7/IL-7 receptor signaling in the development of T lymphocytes, the biological function of IL-7 in the maintenance and survival of T lymphocytes, the performance of IL-7 in pre-clinical and clinical trials of cancer vaccines, and the rationale to apply IL-7 as an adjuvant in cancer vaccine-based therapeutic strategy.

## Introduction

Immunotherapy now is experiencing its “golden age” recently, with the immune checkpoint blocker (ICB) approved by the Food and Drug Administration for the treatment of melanoma and lung cancer, which forever changed the balance in the choice of methods of anticancer therapy (1, 2). The concept of immunotherapy implies antitumor immune response activation or/and immunosuppression inhibition. As one of the critical components of immunotherapy in oncology, cancer vaccines show advantages in specificity, immunogenicity, and low toxicity (3, 4). Unlike ICBs, a passive immunotherapeutic strategy, cancer vaccines possess the capability to induce anti-tumor immune responses actively (5). Moreover, cancer vaccines are time- and labor-saving in manufacturing, when compared to chimeric antigen receptor T cells therapy, making it more feasible for most cancer patients. The current rapid development of the mRNA cancer vaccine brings inspiring results for cancer patients, making this field of research scorching again (6, 7). Although different types of cancer vaccines have been evaluated in clinical trials, few led to satisfying clinical benefits. One of the underlying mechanisms lies in the lack of long-term memory to maintain immune surveillance to prevent the recurrence and metastasis of cancers (3, 8). Increasing studies have revealed that the success of cancer immunotherapeutics, especially cancer vaccines, is critically dependent on the induction of memory T-cell responses against cancers (9, 10). Moreover, the administration of vaccines without immunomodulatory agents is hard to reach the greatest effectiveness to stimulate the antitumor immune response (11). Therefore, searching for adjuvants, which will augment the efficacy of cancer vaccines and contribute to building up a lasting immune memory against cancers, was an attractive issue for researchers.

**Figure 1:**
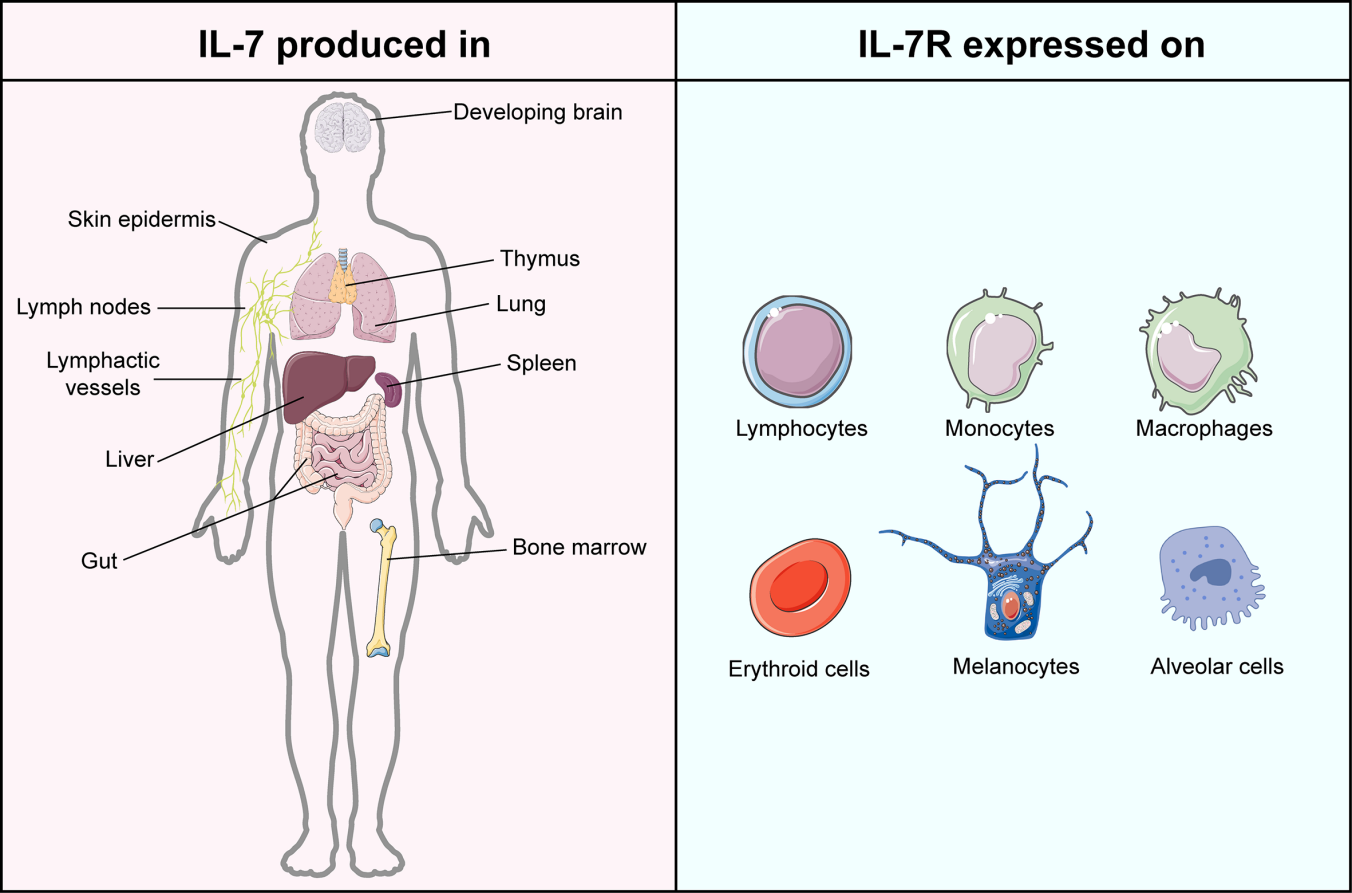
Atlas of IL-7 production and IL-7R expression. IL-7 is mainly produced in but not limited to lymphoid organs, including bone marrow, thymus, spleen, and lymphoid nodes. IL-7 is majorly generated by stromal cells but is also evidenced in epithelial cells, keratinocytes, dendritic cells, and hepatocytes. The expression of IL-7R can be found in lymphocytes, monocytes, macrophages, erythroid cells, melanocytes, and alveolar cells. This figure is created with BioRender.com and smart.servier.com.

IL-7, encoded by the *IL7* gene, is a 25 kDa secreted soluble protein, which was initially discovered by Hunt et al. in 1987 when they explored the latent role of bone marrow stromal cells in the development of the pre-B cell subset (12, 13). Subsequently, increasing evidence proved that except for thymocytes and stromal non-hematopoietic cells (14), IL-7 is also secreted by lymphoid organs, non-lymphoid tissues, and even cancers (15–20) (**Figure 1**). The receptor of IL-7 is a heterodimer complex that comprises an IL-7Rα chain (CD127, encoded by the *IL7R gene*) and a common γ chain (CD132, encoded by the *IL2RG* gene) shared with receptors for IL-2, IL-4, IL-7, IL-9, IL-15 and IL-21 (21, 22). It has been reported that the signaling of IL-7/IL-7R participates in the growth and survival of T and B cell precursors critically (21). Recently, accumulating evidence proved that IL-7 is not only the essential factor in every stage of T cell development (23, 24) but also the crucial component for the survival of naïve T cells as well as the generation and maintenance of memory T cells (25, 26). Furthermore, IL-7 was demonstrated to assist T cells to restore homeostasis *via* the signal transducer and activator of transcription 5 (STAT5)/Suppressor of cytokine signaling (SOCS) pathway (27) and guide T cell homing by inducing the expression of chemokines and integrins (28, 29). In IL-7 knockout mice, a significant depletion of naïve T cells was observed, which can be restored by administrating the exogenous IL-7 (25). Except for T cells, IL-7 was also reported to regulate the immune responses of natural killer cells, dendritic cells, and B cells critically (30–32). As a cytokine therapy for cancer treatment, IL-7 exerts superior activity to induce the expansion of specific T cells against breast carcinoma than IL-2, highlighting its antitumor adjuvant molecular role in oncology (33).

Given the pleiotropic and robust biological effects of IL-7, especially its role in the survival, development, proliferation, and even maintenance of memory of T cells, several research teams apply IL-7 as a molecular adjuvant to strengthen the immunogenicity of cancer vaccines, as well as to maintain a long-term memory response against cancers. Therapeutic cancer vaccines aim to stimulate the immune responses in cancer patients, especially T-cell responses, which have been proven to be the predominant safeguard against tumors. Therefore, in this review, we summarized the biological function and mechanism of IL-7 and the signaling pathway of IL-7/IL-7R in T cells, as well as the performance of IL-7 as an adjuvant in combination with cancer vaccines in preclinical and clinical trials. The rationale to utilize IL-7 as an adjuvant in combination with cancer vaccines was also proposed.

## The biological activity of IL-7 in T cells

### IL-7 in T cell development

**Figure 2:**
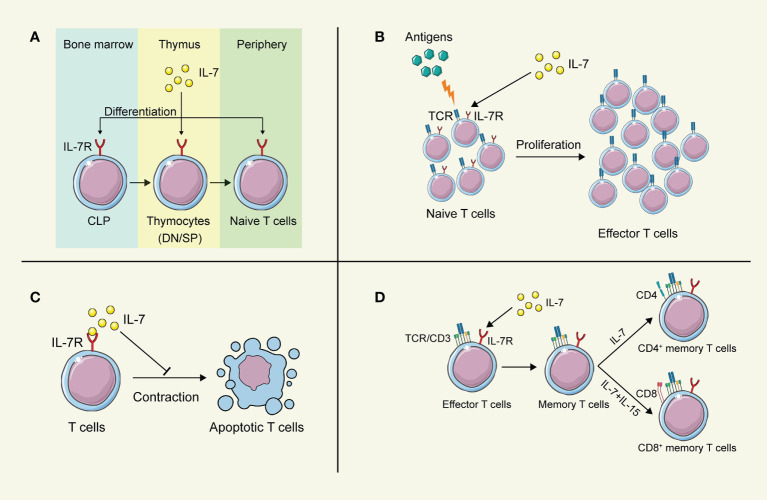
The biological function of IL-7 on T lymphocytes. **(A)** IL-7 contributes to the development of T cells in the thymus. **(B)** IL-7 boosts the proliferation of T cells after stimulation by antigens. **(C)** IL-7 prolongs the survival of T cells. **(D)** IL-7 promotes the differentiation of memory T cells. This figure is created with BioRender.com and smart.servier.com.

Lymphoid progenitors leave the bone marrow and migrate to the thymus for further development into naïve T cells, which were greatly influenced by IL-7 (**Figure 2**). In IL-7 -/- mice, the development and maturation of γδ T cells were restrained significantly, indicating the crucial role of IL-7 in T cell development (34). However, different stages of T cell development in the thymus exhibit distinct demands on IL-7. Pre-T cell progenitors lacking surface expression of CD4 and CD8 are termed double-negative (DN) cells. Based on the amount of CD44 and CD25, DN cells can be divided into four subgroups (DN1-DN4). Development of T cells from DN1-2 thymocytes showed strong dependence on IL-7 (35), while anti-IL-7 antibody led to the deprivation of T cell maturation by interrupting the expansion of T cells at the DN2 stage (36). Differentiation and proliferation of DN3-4 thymocytes also require the presence of IL-7, while the self-renewal of DN4 cells can be substituted partially by the depletion of Bcl6 in the absence of IL-7 (37). In contrast, IL-7 signaling does not participate in the positive selection of CD4 CD8 double-positive (DP), and the IL-7R is not evidenced on the surface of these cells (38). Intriguingly, cells successfully going through the selection express IL-7R again modulated by developmental T cell antigen receptor (TCR)-dependent signals (39).

### IL-7 in T cell survival

IL-7 was initially discovered as a survival factor for mouse T and B cell precursors. It has been demonstrated that IL-7 activates the transcription factor NFATc1 in DN thymocytes *via* phosphorylating Tyr371 to prolong the survival of DN thymocytes, while deficiency in NFATc1 blocked thymocyte development at the DN1 (40). Although DP cells are absent in the expression of IL-7R, SP cells regain it to promote their survival and induce the expression of CXCR4 to enhance the recruitment in secondary lymphoid tissues (41, 42). However, increasing evidence indicates that IL-7 fulfills its regulatory role throughout the lymphoid system. Naïve CD4^+^ T cells consume IL-7 for survival to achieve homeostasis in bone marrow transplant recipients (43). Furthermore, IL-7 also regulates the survival of mature and memory T cells *via* upregulating the expression of Bcl-2 family proteins, leading to long-term memory (44, 45). Additionally, dendritic epidermal γδ T cells show partial dependence on IL-7 for their survival (46). Interestingly, tumor-bearing mice show a decreased level of IL-7 in the spleen, indicating that insufficient IL-7 might fails to support the survival of activated T cells, attenuating the T cell immune responses against tumors (47).

### IL-7 in T cell proliferation

As a highly pleiotropic cytokine, IL-7 provides proliferation signals from hematopoietic stem cells to lymphocytes for their efficient generation (25). At the stage of DN thymocytes, IL-7 appears to promote the proliferation of these cells by upregulating the growth-facilitating genes CD98 in the Stat5-dependent manner (37). After SP thymocytes regain the IL-7R expression, IL-7 still fulfills its capability to stimulate the proliferation of these cells (41). IL-7 facilitates the proliferation of T cells in a concentration-dependent process. A higher concentration of IL-7 stimulates the proliferation of T lymphocytes, while a lower concentration maintains cell survival (48). Administration of IL-7 directly results in an increase in peripheral blood T cells and a broad TCR repertoire diversity (49, 50), while a long-term injection of IL-7 contributes to interrupting the proliferation of T cells, leading to a sharp depletion of naïve T cells (36). Furthermore, the administration of IL-7 in patients suffering from septic syndrome promotes the proliferation of CD4^+^ and CD8^+^ T cells in them, indicating the potential clinical application of IL-7 for septic shock (51). Interestingly, IL-7 was found to antagonize the immunosuppressive network, by abrogating the Treg-mediated suppression (52) and reducing the proportion of Treg cells and myeloid-derived suppressor cells (MDSC) (53, 54), even though IL-7 has little effect on the expansion of Treg cells and MDSCs directly (47).

### IL-7 in the maintenance of T cell memory

After the clearance of pathogens or cancer cells, most of the effector T cells die, and a small proportion of them turn into memory T cells, maintaining the memory against the same antigens (55, 56). During this differentiation process, the critical role of IL-7 is highlighted. Several studies have revealed that IL-7 improves the long-term immune responses against pathogens by inducing naïve T cells to memory T cells (57, 58). A high expression of IL-7R is evidenced in the memory T cells subset, to maintain their survival by upregulating the anti-apoptotic Bcl-2 family proteins (25, 41). The homeostatic proliferation and maintenance of CD4^+^ memory T cells heavily depend on the function of IL-7, while it has been demonstrated that these features of CD8^+^ memory T cells require both IL-7 and IL-15 jointly (59–61). Furthermore, it has been reported that IL-7 is capable to induce more IL-7R^+^ long-living memory stem T cells, which can self-renew and develop into effector T cells (62). Increased administration of IL-7 can boost the specific memory immune responses against cancer and viral infection (45). The adjuvanticity of IL-7 has been verified to prolong the protective effects of vaccines by inducing the production of memory T and B cells (63–65), highlighting the potential to apply IL-7 as an adjuvant in combination with cancer vaccines to struggle against cancer cells *via* long-term memory protection.

## The signaling pathway of IL-7/IL-7R

**Figure 3:**
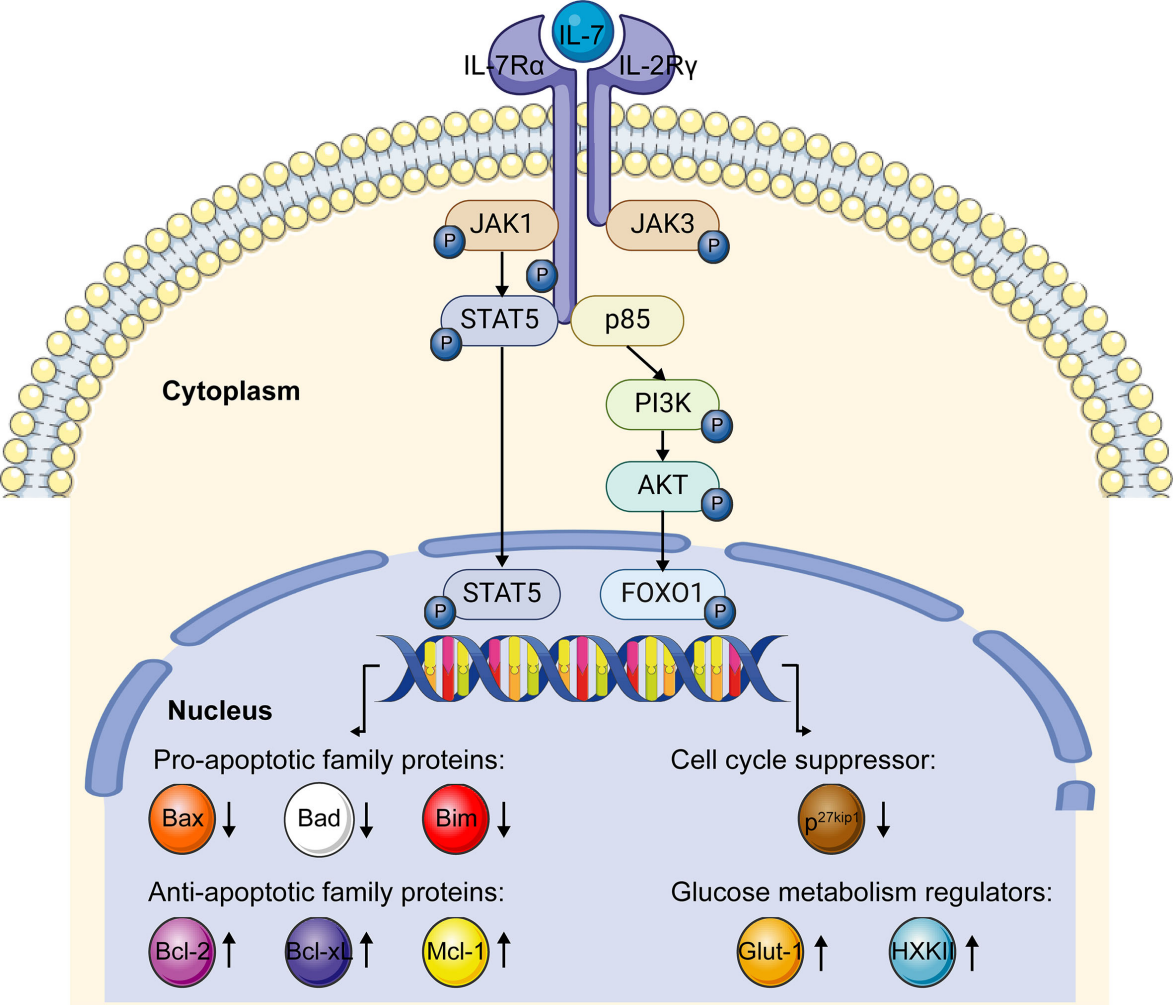
IL-7/IL-7R signaling pathway. When IL-7 interacts with the IL-7R, the α chain and γ chain dimerize. JAK1 and JAK3 were recruited to IL-7Rα and γ chains, separately. After phosphorylation, JAK1 and JAK3 phosphorylate and activate the transcription factor STAT5, which further upregulates anti-apoptotic family genes expression and downregulates pro-apoptotic family genes expression *via* the JAK-STAT5 pathway. After the interaction of IL-7 and IL-7R, recruited p85 induces the phosphorylation of the cytoplasmic tail of the IL-7Rα chain, which further triggers the phosphorylation of the PI3K-AKT pathway. Activated PI3K-AKT signaling induces glucose metabolism regulator genes expression and impedes the expression of cell cycle suppressor p27kip1 *via* FOXO1. This figure is created with BioRender.com and smart.servier.com.

The receptor of IL-7, IL-7R, is a transmembrane heterodimer composed of an IL-7Rα chain and a common γ chain. After the binding of IL-7, IL-7Rα dimerizes with the common cytokine γ chain and triggers kinase activation, while IL-7Rα alone fails to prompt the kinase activity and induce signal transduction (25, 66). The signaling of IL-7/IL-7R is mainly transduced by Janus kinase (JAK)- STAT and Phosphoinositide 3-kinase (PI3K)- Ak strain transforming (AKT) pathways in T cells to fulfill the biological functions of IL-7 (**Figure 3**).

When IL-7 combines with IL-7R, JAK1 and JAK3 are recruited to IL-7Rα and γ chain, and then phosphorylated, respectively. Phosphorylated JAK1 and JAK3 create the docking site for STAT5 recruitment and phosphorylation in an IL-7-dependent pattern (67–69). As a transcription factor, phosphorylated STAT5 dimerizes and translocates into the nucleus, mediating the expression of downstream targeted genes associated with the survival and proliferation of T cells (47, 70). It has been reported that IL-7 prolongs the survival of T cells by upregulating the expression of Bcl-2, Bcl-xL, and Mcl-1 proteins *via* the JAK-STAT signaling pathway (61). Conversely, the inhibitor of JAK activation attenuates IL-7-induced Bcl-2 protein expression (71). Moreover, IL-7 downregulates the expression of the pro-apoptotic genes, like Bad, Bax, and Bim, to prevent apoptosis through the JAK-STAT signaling (72, 73). These studies taken together revealed that the survival of lymphocytes supported by IL-7 is dependent on the JAK-STAT pathway to a large extent.

Another crucial pathway involved in the IL-7/IL-7R signaling is the PI3K-AKT pathway. When IL-7 binds with IL-7R, p85, a regulatory component of PI3K, is recruited and then induces the phosphorylation of tyrosine449 in the IL-7Rα cytoplasmic tail, which triggers the activation of PI3K and then AKT (74). Subsequently, activated AKT participates in the downstream targeted gene regulation by phosphorylating transcription factor the Forkhead box protein 1 (FOXO1) (75). As a targeted molecule of the PI3K-AKT pathway, degraded p27 kinase inhibitor protein 1 (p27kip1) promotes the G1 to S phase transition of T cells in the presence of IL-7, indicating IL-7 can promote the proliferation of T lymphocytes *via* regulating the expression of p27kip1 (76). Furthermore, the PI3K-AKT signaling mediated by IL-7 was reported to regulate the expression of glycolytic enzymes hexokinase II (HXK II) and glucose transporter-1 (Glut-1), to increase the glucose uptake to support the T cells’ survival and maintain homeostasis (77, 78).

## IL-7 as an adjuvant for cancer vaccines in preclinical research

Numerous preclinical trials have evaluated the potential role of IL-7 as an adjuvant to strengthen the effectiveness and long-term responsiveness of cancer vaccines. Here we selectively summarized the performance of IL-7 as an adjuvant in combination with cancer vaccines in preclinical settings (**Table 1**).

**Table 1:** Adjuvant effect of IL-7 in cancer vaccine treatment summarized from selective preclinical studies.

Year	Cancer vaccine	Form of IL-7	Targeted cancer cell lines	IL-7 dose/route	Combination therapy	Study models	Study results	Reference
2021	Recombinant *Mycobacterium smegmatis* delivering a fusion protein of human MIF and IL-7	IL-7 gene inserted in Recombinant *Mycobacterium*	The MC38, LLC, and PanO2 cells	The mice were treated with peritumoral injections of mycobacterium (2×10^6^ bacteria/mice) on days 3, 7, and 14.	Anti-PD-L1 antibody	Seven-week- Old female C57BL/6 mice	Cancer vaccine treatment led to increased activation of CD4^+^ and CD8^+^ T cells in the tumor regions of vaccinated mice, contributing to the antitumor effect. Moreover, cancer vaccine treatment exhibited an enhanced anticancer effect with anti-PD-L1 immunotherapy, in tumor-bearing mouse models.	(79)
2016	HPV DNA vaccine	human IL-7 fused with a hybrid Fc-fragment, which contains the upper CH2 domain of IgD, and the last CH2 and CH3 domains of IgG4	The TC-1 cell line cotransformed with the HPV16 E6, E7 gene	Mice were intravaginally administered with IL-7-Fc (1 mg/kg)	None	8 to 10 weeks female C57BL/6 mice	Topical administration of IL-7-Fc after HPV DNA vaccination increased the quantity of HPV-specific CD8^+^ T lymphocytes in the genital mucosa, resulting in a stronger anticancer immunity than HPV DNA vaccine alone. Mice cotreated with HPV vaccine and IL-7-Fc exhibited significantly attenuated tumor growth and promoted survival rate.	(80)
2016	Whole-cell cancer vaccine coexpressing IL-7 and IL-21	IL-7 gene transfected in tumor cell vaccine	Murine melanoma B16F10 cells and colon carcinoma CT26 cells	Mice were vaccinated s.c. with 1×10^6^ vaccine cells twice at a one-week interval in the prophylactic setting. In the therapeutic setting, mice were treated with two doses of 1×10^7^ irradiated vaccine cells	IL-21	Female C57BL/6 and Balb/c mice	Cytokine production in vaccine cell lines was confirmed. IL-21 and IL-7 co-expressing cancer cell vaccine protected mice from tumor challenges in prophylactic and therapeutic models. Furthermore, the vaccine enhanced the infiltration of CD8^+^ and CD4^+^ T cells in the tumor region. Notably, long-term memory antitumor immunity was demonstrated after vaccine treatment in mice.	(81)
2014	Autologous tumor cell vaccine modified with nonlytic Newcastle disease virus strain LX expressing IL-7 (LX/(IL-7))	IL-7 gene transfected in tumor cell vaccine	The EL-4 murine lymphoma cell line, and B16-F10 murine melanoma cell line	Mice were immunized with 1×10^6^ irradiated cancer cell vaccine LX/(IL-7) s.c. for prophylactic or therapeutic purpose	None	Pathogen-free 6-week-old female C57BL/6 mice	The gene IL-7 product in cancer cell vaccine LX/(IL-7) was active and stable. This vaccine exhibited great prophylactic and therapeutic effects against tumors. Tumor-specific CD8^+^ T cells with higher IFN-γ expression and cytotoxicity were evidenced in models compared to control. However, the percentage of memory T cells was not significantly modified by the vaccine treatment group.	(82)
2009	LCMV, mimicking a live viral antitumor vaccine/*In vitro*–differentiated DCs pulsed with GP33, GP276 and GP61	Recombinant human IL-7	Pancreatic β-islet cell tumors.	Eight days after vaccination, mice received 10 mg of recombinant human IL-7 s.c. daily for 2 weeks.	None	RIP-TAG2 transgenic mice	Survival of IL-7–treated mice was prolonged compared to control mice. IL-7–treated mice with tumors had a 3.5- to 10-fold increase in both CD4^+^ and CD8^+^ T cell numbers while tumors from IL-7–treated mice were heavily infiltrated with both CD4^+^ and CD8^+^ T cells, compared to PBS control mice. Furthermore, Mice receiving DC vaccination together with IL-7 also exhibited an elevated antitumor response.	(83)
2009	Recombinant lentivectors encoding the HLA-A2–restricted Melan-A26-35 peptide	Recombinant human IL-7	None	Mice received intraperitoneal injections of 5 μg human recombinant IL-7 daily after vaccination.	None	HLA-A*0201/H-2Kb transgenic mice	IL-7 adjuvant promoted the proliferation of the Melan-A-specific effector and memory CD8^+^ T cells and enhanced their immune responses after vaccine immunization. The functionality of Melan-A-specific memory CD8^+^ T cells was improved after IL-7 treatment.	(84)
2007	GM-CSF expressing B16F10 and CT26 tumor cell vaccine	Recombinant human IL-7	The B16F10 melanoma and the CT26 colon carcinoma cell	Mouse received 10μg IL-7 administration by s.c. injection, three injections per week for a total of 3 weeks.	GM-CSF	8 to 12 weeks female C57BL/6 mice and BALB/c mice	IL-7 treatment increased the survival of tumor-bearing mice after vaccine administration. IL-7 augmented the number of activated and tumor-infiltrating T cells and specific immune responses in vaccine-receiving mice. Furthermore, a tumor-specific memory response induced by IL-7 protected mice from a second tumor challenge.	(85)
2006	Monocyte-derived DCs pulsed with tumor antigen KLH	Recombinant human IL-7	None	PBMCs were treated with IL-7 in a dose-dependent method	Recombinant human IL-15	PBMCs from patients with metastatic renal cell carcinoma of the clear-cell type	IL-7 induced much stronger proliferation in post- than in prevaccine PBMCs in a dose-dependent manner, while IL-7 induced IFN-γ production in postvaccine PBMCs but not in prevaccine PBMCs. However, a synergetic effect of IL-7 and IL-15 on the proliferation of PBMCs was not evidenced.	(86)

MIF, Macrophage migration inhibitory factor; PD-L1, Programmed death-ligand 1; HPV, Human papillomavirus; DNA, Deoxyribonucleic acid; s.c., subcutaneous; LCMV, Lymphocytic choriomeningitis virus; GP, Glycoprotein; RIP, Rat insulin promoter; TAG2, SV40 large T antigen; HLA, Human leukocyte antigen; DC, Dendritic cell; KLH, Keyhole limpet hemocyanin; PBMC, Peripheral blood mononuclear cell.

**Figure 4:**
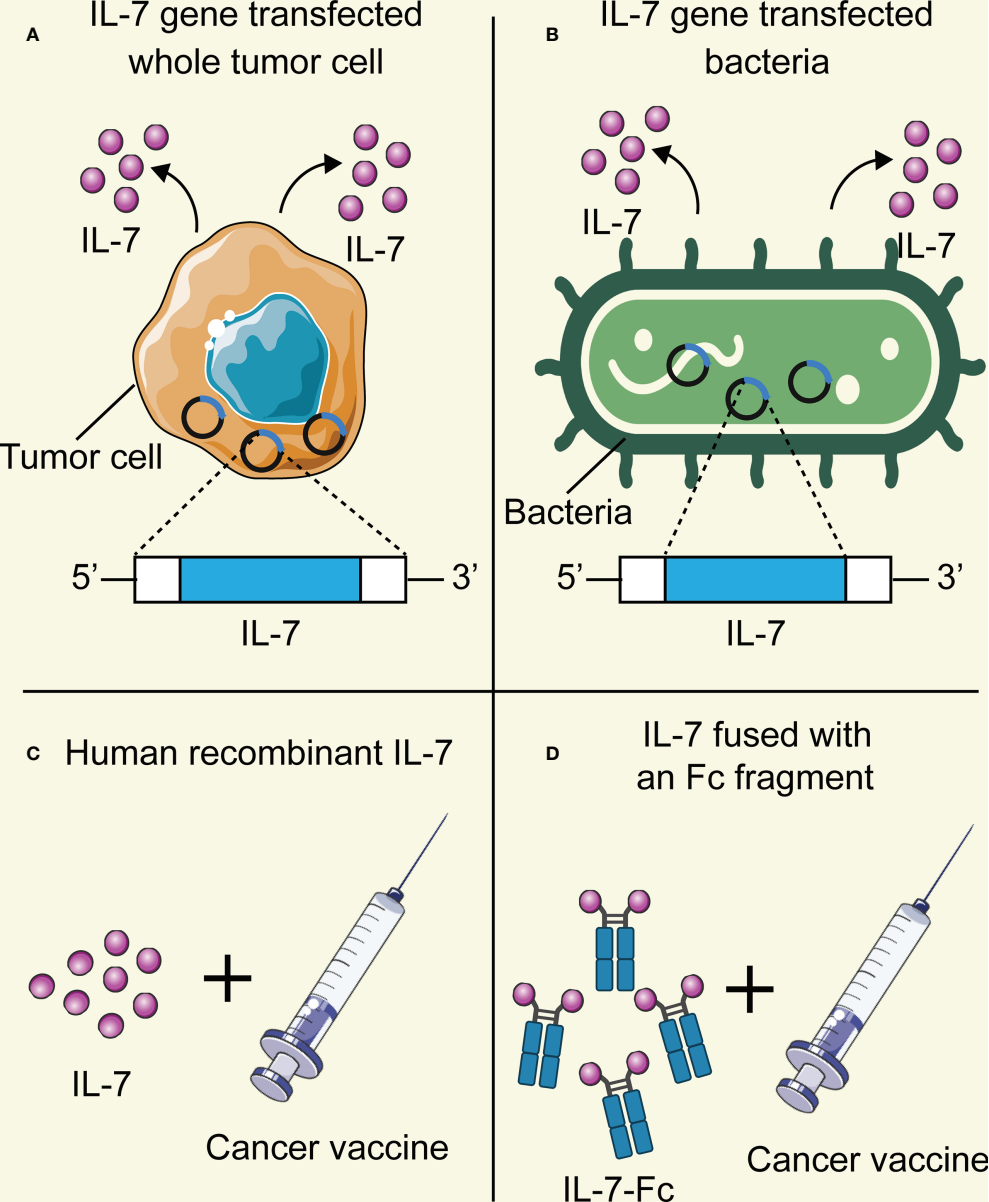
The adjuvant platform of IL-7 for cancer vaccines in preclinical and clinical trials. **(A)** Cancer vaccine tumor cells are transfected with the adjuvant IL-7 gene and then produce IL-7 stably. **(B)** Bacteria are transfected with the adjuvant IL-7 gene and then produce IL-7 stably. **(C)** Direct administration of human recombinant IL-7 as an adjuvant in combination with cancer vaccines. **(D)** IL-7 fused with the Fc region of the antibody as an adjuvant. This figure is created with BioRender.com and smart.servier.com.

Cancer cell vaccines transfected with the IL-7 gene to combat malignant diseases have been extensively explored (81, 82) (**Figure 4A**). Zhao et al. demonstrated that the production of IL-7 in the cancer cell vaccines modified with the IL-7 gene was active and stable in a soluble form, whereas tumor cells as the control settings exhibited an undetectable level of IL-7. Moreover, 10 μl supernatants of transfected cancer cells were equivalent to 1 ng recombinant human IL-7 protein in promoting T cell proliferation (82). Whole-cell cancer vaccines expressing IL-7 showed robust prophylactic and therapeutic effects against cancers, preventing tumor occurrence or prolonging the survival time in mice models. Furthermore, a higher infiltration of CD4^+^ and CD8^+^ T cells in the tumor regions was determined, which was correlated with a better survival outcome in the tumor-bearing mice (81, 82). Except for transfection of the IL-7 gene in tumor cell vaccines, Jeong et al. explored *Mycobacterium smegmatis* delivering a fusion protein comprising human macrophage migration inhibitory factor (MIF) and IL-7 as cancer vaccines to struggle against tumors (79) (**Figure 4B**). This bacterial-based cancer vaccine induced antitumor immune responses by recruiting effective T cells while reducing the infiltration of myeloid-derived suppressor cells (MDSCs) in the tumor environment. Intriguingly, an enhanced antitumor effect of the vaccine was observed in combination with programmed death-ligand 1 (PD-L1) immunotherapy.

However, the most common method to apply IL-7 as an adjuvant to enhance the antitumor effect of cancer vaccines in preclinical research was the injection of recombinant human IL-7 directly (83–86) (**Figure 4C**). Pellegrini and colleagues evidenced that IL-7 treatment in combination with a virus-based vaccine lead to a 3.5-10 fold increase in the number of CD4^+^ and CD8^+^ T cells in tumor-bearing mice, as well as higher infiltration levels of T lymphocytes in the tumor milieu compared with the PBS treatment group (83). Adjuvant IL-7 also contributed to the prolonged survival of activated T cells, enhanced effector responses, and increased production of cytokines, which boost vaccine-elicited immunity and survival in tumor-bearing mice. Furthermore, the same adjuvant effects of IL-7 were demonstrated to promote the efficacy of DC-based vaccines. Intriguingly, Pellegrini et al. also found that IL-7 possesses the capability to antagonize the Treg inhibitory network, which might promote antitumor immunity. The effects of recombinant human IL-7 as an adjuvant in augmenting the number of tumor-infiltrating T cells, specific antitumor immune responses, and survival time of tumor-bearing models also have been revealed in other published studies (84–86). Interestingly, Choi and colleagues novelly fused IL-7 with an Fc fragment to enhance the mucosal delivery across the genital epithelial barrier (**Figure 4D**), aiming at testing the antitumor effects of intravaginal administration of Fc-Fused IL-7 in combination with human papillomavirus (HPV) DNA vaccines against cervical cancer (80). Intravaginal administration of IL-7-Fc induces the recruitment of T cells and various cytokines and chemokines in cervicovaginal tissue, while IL-7 fails to do so. Furthermore, topical administration of IL-7-Fc in combination with the PHV vaccine increased a higher number of HPV-specific CD8^+^ T lymphocytes and exhibited a greater therapeutic effect against cervical cancer. This research highlighted the possibility to apply Fc-fused IL-7 as an adjuvant to strengthen the cellular immunity induced by vaccines in the genital mucosa to struggle against cancers.

However, the adjuvant capability of IL-7 contributing to cancer vaccines was not only limited to inducing a short-term antitumor effect but also stimulating a long-term T cell memory against cancers. Previous studies have reported that cancer vaccines in combination with IL-7 induced a higher proportion of specific CD8^+^ and CD4^+^ memory T cells in mice models bearing tumors and improve the functionality of specific CD8^+^ memory T cells by enhancing their IFN-γ secretion (81, 84). Furthermore, T cell memory elicited by cancer vaccines and IL-7 protected tumor-free mice model from a second tumor challenge (81, 85). Preclinical research highlighted that adjuvant IL-7, when combined with cancer vaccines, contributes to the generation and maintenance of T cell memory, which is the critical component to building up immune surveillance to restrict the recurrence and even metastasis of cancers. Therefore, the adjuvant value of IL-7 for cancer vaccines is further explored in clinical trials.

## The advance of adjuvant IL-7 in combination with cancer vaccines in clinical trials

Preclinical studies have proved that IL-7 could serve as an ideal adjuvant for cancer vaccines to combat cancers. Therefore, based on these preclinical practices, several clinical trials utilizing IL-7 as an adjuvant for cancer vaccines to struggle against malignant diseases were initiated and some encouraging results have been achieved, as summarized in **Table 2**.

**Table 2:** Clinical trials of cancer vaccines combined with IL-7 as an adjuvant for the treatment of malignancy.

Vaccine	Trial ID	Phase	Enrollment status	malignancy	IL-7 dose/route	Timing/length ofIL-7 therapy	Combination adjuvant	Study results	Enrolled population	Reference
Sipuleucel-T	NCT01881867	II	Completed	Metastatic castration-resistant prostate cancer	10 µg/kg/s.c.	IL-7 was given weekly×4 or until the unacceptable AE(s) occurred	None	Treatment with IL-7 was well tolerated. IL-7 led to a significant proliferation of CD4^+^ and CD8^+^ T cells. Increased expression of IL-2, TNFα, IFN-γ, and IL-6 was demonstrated in central memory and effector memory T subsets. No improved PFS or OS in the IL-7 treatment group were observed.	54	(87)
Autologous tumor lysate-pulsed DCs vaccination	NCT00923351	I/II	Completed	Metastatic and recurrent pediatric sarcomas	20μg/kg/s.c.	IL-7 was given on days 0, 14 ± 7 d, 28 ± 7 d, and 42 ± 7 d.	None	No grade 3/4 AEs were reported. An increased number of CD4^+^ and CD8^+^ T cells was evidenced in the IL-7 treatment group. Moreover, IL-7 down-regulates the frequency of regulatory T cells in patients. No difference in OS between subjects treated ± IL-7 was observed.	43	(88)
RCC26/IL-7/CD80 (IL-7/CD80 cotransfected allogeneic renal cell cancer)	NA	I	Completed	Progressive metastatic clear cell RCC	Patients were immunized with 2.5–4.0x10^6^ RCC26/IL-7/CD80, 1x10^6^ RCC26/IL-7/CD80 vaccine cells were able to produce ~4.5x10^3^ pg IL-7/s.c.	2.5x10^6^ cells at weeks 1, 2, 4, and 6; 10x10^6^ cells at weeks 8, 10, 12, and 14; and 40x10^6^ cells at weeks 18 and 22.	CD80 (cotransfected in RCC cells)	Vaccination was clinically safe, with no grade 3/4 toxicities being observed. 50% of the patients showed SD throughout the study and the median time to progression was 18 weeks while the median OS was 40 months. T cell responses were predominantly TH2 type. There was a decline of Treg cells in three patients.	10	(89)
Autologous IL-7 and GM-CSF cotransfected tumor cells	NA	I/II	Completed	Metastatic colon carcinoma, renal cancer, and melanoma	Patients received at least four injections of 1x10^6^ autologous tumor cells transfected with the IL-7 gene/s.c.	Patients received four injections of vaccines on days 0, 14, 28, and 56, respectively.	GM-CSF (cotransfected in autologous tumor cells)	No AEs could be detected in all patients. IL-7 level was elevated in the serum of the patients after treatment. Two patients had SD after treatment while five patients were confirmed with PD. A significant increase in CD3^+^CD8^+^ T cells subset from 21.5 to 25.6% in all patients on day 84 was evidenced. Cytotoxicity of peripheral blood lymphocytes increased significantly during treatment.	10	(90)
MGN1601 vaccine (genetically modified allogeneic tumor cells for the expression of IL-7, GM-CSF, CD80, and CD154)	NCT01265368	I/II	Completed	Advanced renal cell carcinoma	MGN1601 was injected intradermally eight times.	Within 12 weeks.	GM-CSF, CD80, and CD154 (cotransfected in autologous tumor cells)	Administration of MGN1601 to RCC patients was well tolerated. MGN1601-treated patients showed significantly increased OS over the untreated patients. After vaccination, antibodies against TAA were demonstrated in RCC patients.	19	(91)
Melanoma peptide vaccine comprising gp100 antigen and MART-1 antigen	NCT00091338	I	Completed	Melanoma	NA/s.c.	Patients received IL-7 on days 0, 3, 6, 9, 12, 15, 18, and 21.	Incomplete Freund’s adjuvant	NA	NA	NA

IL-7, interleukin 7; µg, Microgram; kg, Kilogram; s.c., Subcutaneous; AEs, Adverse events; PFS, Progression-free survival; OS, overall survival; DC, dendritic cell; RCC, Renal cell carcinoma; pg, Picogram; SD, Stable disease; TH2, T helper 2; PD, Progressive disease; TAA, Tumor-associated antigen; gp, Glycoprotein; MART-1, Melanoma antigen recognized by T cells 1; NA, Not available.

In the early stage, several clinical trials focus on the development of IL-7 gene-modified tumor cells as a cancer vaccine for patients with advanced malignant diseases (89–91). For instance, in a clinical phase I study launched by Westermann and colleagues, they immunized renal cell cancer (RCC) patients with IL-7 and CD80 genes cotransfected RCC tumor cells cancer vaccine (89). This vaccination has been proven to be feasible and safe with no grade III/IV adverse events (AEs) occurring. Throughout the trial, 5 out of 10 patients developed stable disease (SD) and the median time to progression was 18 weeks, while the median overall survival (OS) was 40 months in all. However, vaccination with IL-7 gene-modified RCC cells induced a TH2-predominant but not TH1-polarized immune response against RCC in most patients. In another phase I/II clinical trial, Wittig and colleagues cotransfected IL-7 and granulocyte-macrophage colony-stimulating factor (GM-CSF) genes in autologous tumor cells as a therapeutic vaccine to immunize patients with progressive metastatic carcinoma (90). This vaccine was clinically tolerable with no AEs being observed. Increased level of IL-7 was evidenced in the serum of the patients after treatment. At the endpoint of the study, 2 out of 10 patients achieved SD while 5 remained in progressive disease. The number of CD3^+^, CD8^+,^ and CD56^+^ lymphocytes increased postvaccination. Intriguingly, the cytotoxicity of peripheral blood lymphocytes of patients elevated significantly during treatment, demonstrating that the IL-7-expressing tumor cell vaccine was immunological and capable to stimulate a robust immune response against metastatic cancers.

Recently, some clinical studies attempt to administer recombinant human IL-7 in cancer patients subcutaneously as an adjuvant for cancer vaccines (87, 88). In a phase I/II clinical trial (NCT00923351) (88), recombinant human IL-7 was administered to pediatric sarcoma patients on days 0, 14 ± 7 d, 28 ± 7 d, and 42 ± 7 d after receiving autologous tumor lysate-pulsed DCs vaccination. No grade III/IV AEs were reported during the treatment. Encouragingly, IL-7 recipients showed a higher number of circulating CD4^+^, CD8^+^ T cells, and NK cells than the subjects who did not receive IL-7, exhibiting the great potential of IL-7 to induce immunological reconstitution. Moreover, the administration of IL-7 decreased the proportion of regulatory T (Treg) cells in patients. Regrettably, there was no difference in OS between the patients treated ± IL-7. Another phase II clinical study (NCT01881867) (87), initiated by Pachynski et al., reported that subcutaneous administration of IL-7 resulted in the expansion of CD4^+^, CD8^+,^ and γδ T cells in prostate cancer patients treated with sipuleucel-T, a therapeutic cancer vaccine proved by Food and Drug Administration (92). Furthermore, increased levels of intracellular cytokines were revealed in CD4^+^,γδ T cells, and NK cells. Notably, increased levels of IL-2, TNF-α, IFN-γ, and IL-6 were demonstrated in the memory subsets, indicating that IL-7 possesses the capability to stimulate the memory immune responses against cancers. Although the IL-7 group does show tails of the curves in both OS and progression-free survival (PFS), this trial failed to evidence a significantly improved OS and PFS in the IL-7 arm.

Taking these clinical data together, adjuvant IL-7 exhibited great potential in immunological reconstitution, including prompting expansion of T cells, inducing cytokine production, maintaining memory response, and arousing resistance to immune suppression, when combined with cancer vaccines to struggle against tumors. Moreover, IL-7 was clinically well-tolerant for cancer patients. Unfortunately, the clinical outcome of the patients who received the IL-7 treatment is still unsatisfying, with no enhanced OS or PFS have been demonstrated. However, the population enrolled in these clinical studies is limited. With more subjects included in trials, the survival benefits of the IL-7 arm might be evidenced statistically. More preclinical and clinical studies are encouraged to explore the potential role of IL-7 as an adjuvant for the development of cancer vaccines.

## Proposal of IL-7 as an adjuvant for cancer vaccines in clinical practice

The early-stage clinical trials focus on designing tumor cell cancer vaccines expressing adjuvant molecular IL-7. At least 1x10^6^ IL-7-cotransfected tumor cells were administrated subcutaneously in cancer patients with a minimum of four injections. However, in current clinical trials, direct administration of human recombinant IL-7 subcutaneously as an adjuvant in combination with cancer vaccines is the preferred method. The dose of IL-7 injection varies from 10 µg/kg to 20 µg/kg in cancer patients while at least four doses were administered. Since direct injection of IL-7 as an adjuvant has been proven to be safe and effective, IL-7-cotransfected tumor cell vaccine, which is labor- and time-consuming in manufacturing, seems to be unsuitable and inconvenient in clinical practice. Although direct administration of IL-7 as an adjuvant is more practical, the dose, routine, and time length of IL-7 therapy still need to be optimized according to furthermore clinical trials.

To achieve an effective and long-lasting T cell immune response against cancer to the greatest extent, a combination with other cytokines when utilizing IL-7 as an adjuvant agent for cancer vaccines is recommended. It is crucial to choose suitable chaperone adjuvants rather than a single agent to regulate a full palette of immune responses to combat tumors comprehensively. IL-2 and IL-7 have been well demonstrated to enhance the T cell responses (93), indicating the potential role of IL-2 in combination with IL-7 as adjuvants for cancer vaccines. Another cytokine, IL-15, was also well-defined to boost T cell responses, as well as to maintain long-lasting T cell memory against cancers (94). Intriguingly, antigen-activated T lymphocytes incubated with IL-7 and IL-15 exhibited a more robust capability to induce regression of melanoma and 4T1 mammary carcinoma in comparison to IL-2 alone (33, 95). Moreover, previous studies have indicated that the proliferation and maintenance of CD8^+^ memory T cells require IL-7 and IL-15 jointly (59–61). However, dose-related adverse events of administration of IL-2 and IL-15, like fever and serious biochemical abnormalities in the liver and kidney should not be ignored (96). Furthermore, IL-2 and IL-15 were evidenced to maintain immunosuppressive Treg cells in the periphery and induce the expression of immunosuppressive receptors, resulting in attenuated immune responses (97). To circumvent the immunosuppression induced by combined cytokines, ICB is suggested.

Although the antitumor role of IL-7 in combination with cancer vaccine has been revealed, there is still a “dark side” that needs to be emphasized. IL-7 was evidence to promote cell viability, cell cycle progression, and growth of T-cell acute lymphoblastic leukemia (T-ALL) *in vitro* (98). Moreover, *in vivo* experiments exhibited that the consumption of IL-7 contributes to the development of T-ALL, while IL-7 deficiency reduced the proliferation of leukemia cells (21, 99). Coincidently, IL-7 can also induce the expansion of B-ALL cells (100) and boost the tumorigenesis of B cells in IL-7 transgenic mice (101). These collected facts highly indicated that IL-7 acts as a tumor-promoting factor during the occurrence and progression of hematopoietic malignancy. Therefore, it is unpracticable to apply IL-7, a cytokine that contributes to leukemia development, as an adjuvant for cancer vaccines against hematopoietic malignancy for safety considerations.

## Conclusion

Various factors affect the immunogenicity of cancer vaccines, like the selection of antigens (102), the choice of adjuvant (103), and even the delivery vehicles that are applied in the administration strategy (104). To stimulate an effective and long-lasting T cell memory response, cancer vaccines incorporated with adjuvant IL-7 to combat cancers were initiated in numerous experiments. The biological role of IL-7 in the development, survival, proliferation, and memory maintenance of T cells was summarized while the signaling of IL-7/IL-7R was summed up in this manuscript. These facts highlighted the feasibility to apply IL-7 as an adjuvant in combination with cancer vaccines. Preclinical researches bring inspiring results for the rationale to apply IL-7 as an adjuvant for cancer vaccines while further clinical trials enhance this notion. Although boosted T cell expansion and enhanced T cell memory were evidenced in clinical trials when administrating IL-7 as an adjuvant combined with cancer vaccines, the clinical outcomes of cancer patients are still far from satisfactory. Currently, it is still hard to give a conclusion that IL-7 can serve as an ideal adjuvant for cancer vaccines from such a limited number of clinical trials, therefore, clinical trials concerning IL-7 as an adjuvant for cancer vaccines, to fulfill efficacious T cell responses and long-lasting T cell memory against tumors, are urgently encouraged. Here, we also proposed some suggestions on how to utilize IL-7 as an adjuvant incorporated with cancer vaccines, including the form, dose, and chaperone cytokine of IL-7, which might be of value to researchers.

## Author contributions

XL and ZX contributed to the study conception design. YZ, KW, and HC performed the literature search and data collection. Figures were drawn by YZ. YZ and KW prepared the draft and the manuscript was revised by XL and ZX. XL provided the funding. All authors contributed to the article and approved the submitted version.

## Funding

This study was funded by the National Natural Science Foundation of China (grant number 81871653), the Natural Science Foundation of Chongqing (cstc2021jsyj-yzysbAX0018), Chongqing Science and Health Joint Medical High-end Talent Project (2022GDRC012), Science and Technology Research Program of Chongqing Municipal Education Commission (KJZD-K202100402, KJQN201900449) and CQMU Program for Youth Innovation in Future Medicine (W0073).

## Conflict of interest

The authors declare that the research was conducted in the absence of any commercial or financial relationships that could be construed as a potential conflict of interest.

## Publisher’s note

All claims expressed in this article are solely those of the authors and do not necessarily represent those of their affiliated organizations, or those of the publisher, the editors and the reviewers. Any product that may be evaluated in this article, or claim that may be made by its manufacturer, is not guaranteed or endorsed by the publisher.
